# Quick beginners guide and tips on how to write a manuscript

**DOI:** 10.1590/S1677-5538.IBJU.2020.05.02

**Published:** 2020-07-31

**Authors:** Leonardo Oliveira Reis

**Affiliations:** 1 Universidade de Estadual de Campinas UroScience, Departamento de Urologia Campinas SP Brasil UroScience, Departamento de Urologia, Universidade de Estadual de Campinas - Unicamp, Campinas, SP, Brasil; 2 Pontifícia Universidade Católica de Campinas Disciplina de Urologia Campinas SP Brasil Disciplina de Urologia, Pontifícia Universidade Católica de Campinas - PUC - Campinas, SP, Brasil

## COMMENT

Do you have a piece of work that might be appreciated by many experts all over? Make sure it was Ethics Committee appreciated and just take a weekend of work and make a competitive manuscript, the very first step to get published.

If you really understand something, you must be able to explain it in an easy way, and that is our challenge when compiling experience on “how to write a manuscript” without overkilling yourself.

This guide will make possible to easily transform your “idea”, study, thesis in a science “brick” or “cell” as we are more at the biological side, as the smallest structural and functional unit of the whole body of knowledge on every topic, accessible to other scientists all over the globe.

## TO THE POINT

TITLE: Straight message that hooks the reader. Can be a disrupting question or even an answer. Be creative - it really matters.

ABSTRACT: a mini manuscript with the essentials in structured 250 words. It is easier to make it unstructured in case you need, than the opposite. Purpose/Methods/Results/Conclusions.

## MANUSCRIPT OVERVIEW

In the Surgery/Urology field usually 3000 words is the rule, distributed in the percentages below:

INTRO: 10 % - 3 paragraphs right to the point - not a discussion, never longer.

METHODS: 20 - 30 % - may vary according to the methodology density/complexity.

RESULTS: 20 - 30 % - may vary according to results density/complexity.

DISCUSSION: 20 - 30 % - perspective related to the literature.

CONCLUSION: 1 paragraph, just a message, maybe the Discussion last paragraph.

## MANUSCRIPT IN DETAIL

INTRODUCTION: 3 paragraphs (less is more - introduction is not a discussion).

Paragraph 1- Shows the issue relevance / impact - prevalence, morbidity, mortality.

Paragraph 2- Identifies the “GAP” or what is unknown (the study Justification).

Paragraph 3- Fills de “GAP”, usually describes the study hypothesis/objectives.

METHODS: usually 6 to 9 paragraphs.

Like in a cake recipe the reader should be able to replicate your study. Describe in a chronological sequence, keep to the essential steps. Rely on previous papers and keep to brief descriptions. Everything you have before putting the plan in practice belongs to methods.

RESULTS: usually 6 to 9 paragraphs.

Everything you obtain after putting in practice what were planned belongs to results. Tables and figures illustrate the story (complement never repeat). A chronological description is usually adequate.

Initial results are usually those that characterize the environment, the cohort and is essential to define study representativeness and applicability, before showing what was found in fact (i.e. Table-1, demographics). Just show data - never discuss in the results section. If too much data, think about organizing in different scopes, making more than one paper.

DISCUSSION: usually 6-9 paragraphs.

Paragraph 1: Describe the message in your results. While in the RESULTS section you showed the numbers, tables, graphics, p values, etc.; you will now give it an interpretation, a description of what you found: what increased, decreased, kept stable? How strong/ big was it? Then develop the following:

How the study/results interact with what is already published? In which aspects it confirms (or which studies your data supports), confronts (which studies your data might refute) or adds to previous data/studies (in which aspects it is new)?

Last Paragraph: Recognize the study limitations and show clinical implications, future perspectives.

CONCLUSION: 1 Paragraph: Expand the title... to give your message. Obviously supported by your results only. Avoid overstatements (don’t tell what was not showed/ supported by your results).

REFERENCES: Have read the best evidence available on the topic you are writing and keep the essential and UpToDate works as references. Avoid using review articles cause might bypass the real authors that built the “bricks” you might be using.

## LAST WORDS

Keep the habit of reading papers, looking deep in their skeleton or structure and doesn’t matter how much experience you have, just follow the above-mentioned steps and put your study or thesis in perspective. Usually less is more and every assumption you make must be substantiated by facts/data (1). Know that your audience is made of editors, reviewers, readers, scientists and remember, the main challenges in this game are to:

Write a clear, easy, informative and enjoyable text.

Be honest and transparent.

With that you will convince the editor and reviewers about the importance of your manuscript and hook the reader’s attention with your published paper, elevating the chances of acting as one fundamental “brick” in the wilderness of scientific building.

In this enjoyable process you will perceive that paper structures vary with hyper- and hypotrophy segments and eventual “appendices” according to the strategy/methodology. When you become proficient in seeing the papers’ skeleton through the “soup of words”, is time to the next step, the quality control by using specific reporting guidelines, checklists and quality control according to the study type at <https://www.equator-network.org/> (2).

Good luck, respect others ideas, believe in yourself, and remember, beyond the academic arena resilience is one of the most important qualities.
